# Patient Engagement in Oncology Practice: A Qualitative Study on Patients’ and Nurses’ Perspectives

**DOI:** 10.3390/ijerph191811644

**Published:** 2022-09-15

**Authors:** Angela Tolotti, Serena Barello, Camilla Vignaduzzo, Sarah Jayne Liptrott, Dario Valcarenghi, Tiziana Nania, Davide Sari, Loris Bonetti

**Affiliations:** 1 Nursing Development and Research Unit, Oncology Institute of Southern Switzerland, Ente Ospedaliero Cantonale (EOC), Via Gallino, 12, 6500 Bellinzona, Switzerland; 2 EngageMinds HUB—Consumer, Food & Health Engagement Research Center, Department of Psychology, Università Cattolica del Sacro Cuore, Milano and Cremona, L.Go Gemelli 1, 20123 Milan, Italy; 3 Nursing Development and Research Unit, Regional Hospital of Bellinzona and Valli, Ente Ospedaliero Cantonale (EOC), Via Ospedale 12, 6500 Bellinzona, Switzerland; 4 Health Professions Research and Development Unit, IRCCS Policlinico San Donato, Piazza Edmondo Malan, 2, San Donato Milanese, 20097 Milan, Italy; 5 Department of Nursing, Oncology Institute of Southern Switzerland, Ente Ospedaliero Cantonale (EOC), Via Gallino, 12, 6500 Bellinzona, Switzerland; 6 Nursing Research Competence Centre, Department of Nursing, Ente Ospedaliero Cantonale (EOC), Viale Officina, 3, 6500 Bellinzona, Switzerland or; 7 Department of Business Economics, Health and Social Care, University of Applied Sciences and Arts of Southern Switzerland, Via Violino, 11, 6928 Manno, Switzerland

**Keywords:** patient activation, empowerment, patient engagement, patient involvement, patient participation, neoplasm, nurse, qualitative research

## Abstract

Patient engagement has gained increasing attention in cancer care as it is widely acknowledged as an essential element of high-quality care. There are limited data on how oncology nurses might apply techniques that encourage patient engagement. Therefore, this study aims to understand which nursing strategies can favour patient engagement in oncological care from patients’ and nurses’ perspectives. We conducted a qualitative study involving oncology patients and oncology nurses. Patients were interviewed, while nurses were involved in focus groups (FGs). Both interviews and FGs were analysed by the means of thematic analysis. We interviewed six patients and conducted two FGs, involving 17 nurses. Five themes were identified from patients’ interviews: effective information, having the opportunity to choose, being considered a person, trusted relationship with nurses, and receiving support and advice. Additionally, five themes were identified from the FGs: the nurse–patient relationship, personalisation of care, information style, engagement strategies, and the team. The participants highlighted the importance of comprehensive information in order for patients to feel more involved. Great importance was given to the nurse–patient relationship, which must be based on trust and mutual respect. Both nurses and patients emphasised the importance of person-centred care. A more systematic implementation of suggestions from the participants in this study is desirable for the future.

## 1. Introduction

Patient engagement has gained increasing attention in cancer care as it is widely acknowledged as an essential element of high-quality care [1]. Legislative changes, healthcare reforms, and policy requirements in the healthcare sector are driving patient engagement efforts because they serve the objectives of worldwide healthcare systems to enhance treatment quality, reduce costs, and enhance population health [2,3]. Patient engagement generally refers to the delivery of information by healthcare systems and the recognition of individual preferences to empower patients to take action and make choices that will maximise their benefits from the received care [4]. Moreover, patient engagement is a marker of the patient’s ability to be psychologically resilient to the illness experience and, thus, to be an effective owner of his/her own health journey after the diagnosis [5]. Finally, the engagement of the patient can aid in decision-making and behavioural modifications that improve health-related outcomes, minimise the costs of treatment, and improve patient satisfaction [6,7,8].

In this scenario, nurses contribute significantly to engaging patients in the care process through their continuous interaction with patients. Previous studies have demonstrated that nurses have a pivotal role in educating, encouraging, motivating, and supporting patients to be engaged in their own care and to achieve their health care goals [9,10,11,12,13]. To improve health outcomes, patients need to be engaged in reaching these goals, and nurses have a pivotal role in reaching this aim. Therefore, mapping and fostering nurse-related patient engagement strategies is of vital importance when training these professionals for this goal [14].

There are limited systematic data on oncology nurses’ behavioural techniques and strategies that encourage patient engagement, even though a number of stakeholders have advocated for patient-centric health care change. Hence, to find nursing practice-based behaviours and strategies for patient engagement in oncology, we conducted a qualitative study with both nurses and patients. The authors of this project decided to conduct qualitative research because, to the best of their knowledge, just a few studies have analysed what kinds of nursing behaviour might foster patient engagement. Therefore, because it is a relatively novel area of study, we found a qualitative approach more useful in understanding in depth the point of view of patients and nurses on this topic. To reach this goal, the following topics were examined from the viewpoints of both patients and nurses: (1) attitudes toward patient engagement; (2) the components of patient engagement; (3) the main obstacles to implementing patient engagement in daily practice; and (4) strategies that improve patient engagement. These are crucial knowledge gaps that need to be filled in order to properly train and sustain these professionals, especially given the difficulties involved in systematically incorporating and promoting patient engagement into daily healthcare practice.

For the current paper, we focus predominantly on patient engagement in terms of patient-provider–healthcare team interaction, but we acknowledge that patient engagement is broader and includes additional areas of focus at the organisation and policy levels.

### 1.1. Design

This is a qualitative description [15] that has been conducted through individual in-depth interviews with patients and focus groups with nurses. It is a part of a wider study, aimed at developing a checklist to assess what kinds of nurse behaviour increase cancer patient engagement. A qualitative description is useful when the researcher is interested in a straightforward description of a phenomenon or in developing a new tool or questionnaire, which was the aim of the wider study [15]. Furthermore, a qualitative description does not follow a specific qualitative philosophy or method, such as phenomenology, ethnography, or grounded theory. We also decided to adopt this approach because our research focus was not on experience, perception, cultural or social dynamics. Therefore, a more open qualitative approach was more suitable in our case. 

### 1.2. Setting

Two oncology wards (a haematology unit and a radiotherapy unit) and four inpatient oncology departments of the Oncology Institute of Southern Switzerland (IOSI).

### 1.3. Researchers’ Characteristics

Almost all the researchers were experts in qualitative research, with several publications in peer review journals. They did not have a direct relationship with the participants because they did not work in the clinical setting involved in the study. Therefore, they were not influenced by knowledge of or existing relationships with the participants during data collection. Although some authors were experts on patient engagement, the data analysis and themes identification were always done by two researchers independently and discussed among the research team members. Therefore, a possible influence by previous knowledge of the topic was prevented. 

### 1.4. Participants

#### 1.4.1. Patients

Patients were selected among the ones in care during data collection in the two oncology wards (a haematology unit and a radiotherapy unit) and four inpatient oncology departments of the Oncology Institute of Southern Switzerland (IOSI). 

#### 1.4.2. Inclusion Criteria

- All cancer patients able to speak and understand the Italian language;
- Nurses working in the two oncology wards and the four inpatient departments;
- Nurses working within the oncology setting for a minimum of 6 months.

#### 1.4.3. Exclusion Criteria

- Patients receiving palliative care;
- Paediatric patients;
- Patients with dementia, cognitive impairment.

#### 1.4.4. Nurses

- A focus group sample was constructed to ensure maximum variability with respect to socio-demographic characteristics, such as gender, age, and work experience, in oncological care. The only exclusion was an underage person doing work experience in oncological care for six months.

### 1.5. Data Collection

Semi-structured interviews and focus groups were conducted to further explore the patients’ and nurses’ perceptions about the strategies that were felt to be more effective in promoting patient engagement. A sample of patients was constructed to ensure maximum variability with respect to socio-demographic characteristics, such as gender and age group, based on the characteristics of the care pathway. Patient recruitment continued until data saturation was achieved.

The interviews were conducted by two researchers who are experts in qualitative methods in a quiet, dedicated room without external interference. 

The focus groups were conducted with nurses who were working in the inpatient wards involved in the research.

Patients’ interviews were conducted before the focus groups because the patients’ interview themes were used to adapt the focus groups’ guides.

The patients’ interviews and focus groups’ guides are reported in Supplementary Material S1 (S1).

Participation in the study was voluntary. Both the patient interviews and focus groups were audio-recorded and transcribed verbatim.

## 2. Data Analysis

Both the patient interviews and focus groups with nurses were transcribed, and the transcription was repeatedly read, followed by the conduction of a line-by-line thematic analysis [16,17]. The analyses were conducted by expert qualitative researchers (A.T., S.J.L., S.B., C.V. and L.B.). The analysis was data-driven and based on the participants’ unique perspectives rather than guided by a pre-defined theory or hypothesis. Investigator triangulation was used to validate the findings. All researchers read and re-read the transcripts to gain a sense of the content and an overview of the material. With the aim of the study in mind, the researchers highlighted text and made notes and headings in the margins to include all aspects of the content. Initial thoughts and impressions regarding the material were written down. No pre-defined structures were used as the codes were derived from the data to capture key concepts. Codes that were related to each other were grouped and organised into subcategories and categories. This process was iterative, going back and forth and checking the codes against the material. The subcategories and categories were subsequently compared for differences and similarities, with the aim of being as internally homogeneous and externally heterogeneous as possible. An example of the data analysis process is reported in Supplementary Materials S2 (S2).

### 2.1. Rigour

The methodological rigour of the data analysis followed the criteria described by Guba and Lincoln [18]: credibility, transferability, dependability, and confirmability. In order to achieve credibility, interviews were conducted by the same researcher, an expert in qualitative data collection procedures and in-depth interviews. Transferability was ensured by providing a detailed description of the study procedures. Dependability was ensured by the provision of direct quotes for each theme. Finally, confirmability was addressed by the independent analysis of interviews and focus groups by two researchers and subsequent comparison with a third researcher, who was consulted in the case of disagreement [18,19]. The final themes were then discussed among the research team members.

### 2.2. Ethical Issue

The study was conducted following the Helsinki guidelines for human research. The protocol of the study was approved by the regional ethical committee. All participants signed informed written consent forms for audio recording. Anonymity and privacy have been granted.

## 3. Results

Data collection started with the interviews in January 2021 and ended in June 2021 with the second focus group.

### 3.1. The Interviews

Six interviews were conducted with cancer patients aged between 43 and 84 years old with different oncological diagnoses (Table 1).

From the analysis of the interviews, five main themes were identified regarding the meaning and attributes of patient engagement in cancer care: “*effective information*”, “*having the opportunity to choose*”, “*being considered as a person*”, “*trusted relationship with nurses*”, “*receiving support and advice*” (Supplementary Materials (S2)).

Theme 1: Effective information

This theme groups three sub-themes: To know is to understand, to be engaged through information, the right time and the right content.

Information plays a fundamental role in enabling people to understand their own health situation and is the first step towards being involved in the care process. This is something that is considered very important by the interviewees. Information also allows people to understand what is going to happen, what path they will have to face, what therapies are available, and what benefits or risks are involved.

*“...in my opinion it’s very important. In the sense that at least you make a patient aware of what’s going to happen, of what I’m going to have to do, I’m going to have to endure or not … making me part of the situation, of the evolution of the situation, of what could be the problems … that is, of the whole treatment pathway”*

(Male, aged 58)

People expect to be engaged through complete information, and when information is lacking, people do not feel engaged.

*“I expected that maybe there would be more involvement, saying like today we’re going to irradiate this, today we are going to irradiate this other, we’ve finished with this part and we’re going to start with the other. The only information I was given by the Doctor was that the first part of the treatment lasted 20 days or 25, was about the lymph nodes that had already formed, bombard the different lymph nodes in the surrounding areas. And the last 15 sessions I think, 15 or 14, for the prostate directly […] if I’d have had some more information, it would increase my involvement. We need information!”*

(Male, aged 84)

Another aspect that is considered important is related to the type of information and the moment in which it is provided, i.e., knowing how to choose the information that is useful at that moment for the person is important.

*“They always treated me very well, motivated, said the right things at the right time eh…very positive experience …”*

(Female, aged 43)

Questions also play an important role in feeling involved in the care process. Having a space in which one feels free to ask questions and receive answers is crucial. People also appreciated it when nurses, faced with a question they did not know the answer to, inquired and provided the answer.

*“if I have some questions to ask, they know how to answer me, they go and ask the Doctor […] then if I’m at home and I need to ask something, I just call here and they know how to answer too”*.

(Female, aged 72)

Theme 2: Having the opportunity to choose 

This theme groups four sub-themes: the role in decision-making, lack of information, to revisit what has been said, and trust in nurses.

Being able to choose (being able to decide) is a very relevant issue for patients. A cultural aspect related to decision-making power is highlighted. Some patients narrated how, today, there is a greater possibility of being able to decide, whereas, in the past, this power was delegated to the doctor.

*“Nowadays we’re lucky enough to be able to decide, it used to be done and there was little to say. So if I don’t like it, I tell him what I think”*

(Female, aged 65)

Some patients wished to be able to decide, but a lack of information prevented them from doing so. The issue of information is closely related to being able to decide.

*“Maybe say ok, the treatment we propose is this one […] but for how I tend to see things, I wouldn’t have minded having a couple of things on which I could base my choice”*.

(Female, aged 43)

On the contrary, some people thought that it should be the doctor who plays an important role, not only in proposing the course of treatment but also in choosing the treatments. The reason they attribute the decision-making role to him lies in his competence. The nurse is seen as an important figure. The presence of the nurse next to the doctor allows the person to revisit what has been said with the nurse, who will help patients understand what has been decided.

*“Ehm yes, the doctor, he… everything, that’s it and so and so he comes there, makes his diagnosis, he says so and so, and then afterwards, basically, the nurse that is with him takes it in and after helps me, that’s it I’m more in contact obviously with the nurse”.*

(Female, aged 65)

Trust is an element that people have reported as a reason for delegating decisions about their pathway to the doctor and/or nurse. This trust is based on both the competence they recognise in professionals and the information these professionals provide about the actions they are about to take. Trust is also based on the belief that the nurse will carry out what the doctor has decided, a decision that is not questioned.

*“They (the nurses) yes yes, they explain to you, let’s do this, let’s put this on, that on. Do what you have to. And that’s what trust is all about, because they know everything, what’s it got to do with me? I don’t have to be the doctor. […] and so I say, ok, ok and so I’m there calm. They obviously have the doctor’s indications. Alright I think. So, and so it’s fine.”*

(Female, aged 72)

Theme 3: Being considered a person

This theme groups two sub-themes: not feeling like a number and being seen as a person.

Some people highlighted the importance of not feeling like a number, which happens in large hospital settings, where one feels like a stranger or an unknown person. Being seen as a person is important for several reasons: it allows one to heal, it gives one energy, one does not feel invisible, and one feels understood.

*“Here you’re not a number you’re a person, it’s really important this, very important, the most beautiful thing there is this. […] why is it important? Because it’s one of the systems to be able to heal, there is the understanding of the other that’s fundamental, it’s almost half the treatment in my opinion, to give me the energy, it’s really important …it also gives me that buzz, it’s important you know? The mind, to overcome an illness it’s important.”*

(Male, aged 54)

One factor that allows the feeling of being considered as a person is the interest that nurses show in people, not only regarding their illness but also regarding all aspects of their life.

*“Once I was hospitalized, in another ward for the same problems, the nurse and the doctor came to find me “so how are you doing?” and they asked about me and I was really happy about that.”*

(Female, aged 72)

Not feeling like a number within the healthcare setting and the attention that nurses give patients converge to a personalisation of care.

*“They even accommodate me where I want to go in the room, because they know that I like to be towards the window lying down, and I know that I can ask them […], I mean, knowing that there are people that care for you, and for me it’s essential, because otherwise you just become a number.”*

(Female, aged 72)

Theme 4: Trusted relationship with nurses

This theme groups two sub-themes: the nurse is a co-actor in the patient care process and feeling that the nurse is interested.

The nurse is regarded by patients as an important reference figure, a co-actor in their care process, and a person whom they trust and to whom they surrender themselves.

*“The nurse is fundamental because there is always that direct relationship. The doctor has a whole other outfit, right? But with the nurse there is a direct relationship, so it’s essential that ... she’s the point of reference, really a person to whom you can surrender yourself and say here you are now she’ll help me and it’s.… I can let myself go, just say I have great trust.”*

(Female, aged 43)

In the relationship with the nurse, people have narrated how important it is to feel their interest and to be listened to when establishing a trusting relationship. The time devoted to the relationship is an important element, and it is often considered to be insufficient.

*“They are very technical, very precise, but also very human and they are able to listen and that’s really nice, I don’t like coming to this place, but knowing that there are these people that welcome me with a smile on their face is something …beautiful. And I also find them very competent whatever I ask, if they don’t know, they go and ask the doctor, here and there, and I trust them completely”*.

(Female, aged 72)

Theme 5: Receiving support and advice

This theme groups two sub-themes: helping people to cope with their difficulties and spurring the person on.

The interviewees reported having had some moments of discouragement in their journey, and, in these cases, they appreciated the support and encouragement provided by the nurses, which proved to be a key weapon in helping people to cope with these difficult moments. The support provided by the nurses was highly appreciated; it was something that gave the patients strength.

*“When one is a bit down, a push does you good. […] now let’s see if we can sort things out and ask your husband to take you out a bit, to do that, to do the other, like practical things […] because, well, she said to do this too, to go for a walk, to go out for a bit for example, instead of being down, and for me that was fundamental”*.

(Female, aged 72)

Besides the support provided by the nurses, people also appreciated the advice they received. This advice covered a variety of topics, from treatment to diet, sometimes in the form of encouragement to spur the person on to continue the path undertaken.

*“Every time I come here they give me advice […] now would it be better to do a bit of physiotherapy? And, yes maybe it’s better to have some physiotherapy and then a bit you go to the physio and a bit you go out”*.

(Female, aged 72)

### 3.2. Focus Groups

Two focus groups were conducted with nurses. Nine nurses participated in the first focus group and eight in the second. The demographic characteristics of the participants are reported in Table 2. 

As emerged from the interviews, the themes identified cut across the three dimensions of the patient engagement experience according to Graffigna and Barello [5]: cognitive, emotional and behavioural.

Five themes were identified regarding the meaning and attributes related to patient engagement from the nurses’ perspective: “*trusted nurse–patient relationship*”, “*personalisation of care*”, “*information style*”, “*engagement strategies*” and “*the care team as a patient engagement catalyser*”.

### 3.3. Trusted Nurse–Patient Relationship

It is a fundamental prerequisite for patient involvement. It is a relationship that is based on trust and that is made possible through several elements. The first element that the nurses have reported is active listening. Nurses believe that the relationship of trust they establish with the patient through active listening is something exclusive to and strictly within the nursing profession and does not belong to other professions. They emphasise the difference between communicating and listening, and they think that, sometimes, true listening does not take place. Active listening also involves the nurses’ ability to show the person their intention and interest in listening.

*“Don’t stop at the door, that’s it, these are all behaviours that show that you are there and that you’re ready to listen to him, and I would say to show you are sincerely curious about what he is saying to you, maybe “can you tell me again, I didn’t understand it well” I don’t know, about what he said to you. These are all things that afterwards you show him that you are genuinely interested in what he is saying to you and encourage him to talk and open up”*

(Nurse 7)

The environment and the language used can also facilitate the building of a trusting relationship. Paying attention to the setting; avoiding interruptions in communication means taking care of the communication process with the person. The language used can also be helpful in reducing possible communication distances between the nurse and the individual.

*“You have to create a safe, protected environment where there isn’t a bustle of colleagues, without interrupting the patient too much because when you start interrupting too many times, something breaks away”*

(Nurse 10)

Showing availability, making the person feel free to speak or not, being able to express him/herself freely and guaranteeing the confidentiality of what s/he communicates are other necessary elements for building a relationship of trust. Maintaining continuity in the relationship with the person is also fundamental, as is leaving them free to choose the person with whom they wish to share their own or their family members’ feelings and emotions.

*“It’s having as much continuity as possible, as they were saying before, both on an outpatient and inpatient level, we try a little bit to follow the patient often so that we can establish this relationship of trust because if he keeps changing people it’s clear that this is more difficult.”*

(Nurse 8)

Two other important elements in the relationship with the person are knowing how to welcome their emotions and not blame them. The emotions that the person feels are present in various forms during their treatment, and it is important to take them into account. Sometimes the person may be non-compliant, and, in these cases, it is very important not to blame them but to welcome their difficulty.

*“Don’t blame […] if you blame him or even if he only perceives it that way, that the team is talking behind his back and maybe is talking about this non-compliance, they tend a little bit to close themselves off, they tend to do worse or not to do it, not to do it just to avoid doing it wrong”.*

(Nurse 2)

### 3.4. Personalisation of Care

Nurses emphasise how important it is to personalise the individuals’ care pathway, considering the person in front of you and their personal and social situations. Active listening makes it possible to understand whether the patient can cope with the proposed pathway or whether he or she has difficulties that could reduce compliance. Not being rigid but flexible, constantly adapting one’s actions, and considering the person’s needs and what is possible for them means involving the individual in finding satisfactory answers to the problems they encounter together.

*“A patient with a very important oral mucositis who had had chemotherapy, a chemotherapy that gives kidney damage, so you have to hydrate. We usually say to patients “you mustn’t drink fizzy drinks and don’t drink drinks that contain for example, I don’t know, lemon”, well this patient wasn’t able to drink if it wasn’t a fizzy drink. She put up with it, it didn’t bother her. She showed me the solution because I could have continued to tell her to drink water, but it didn’t work, but she took this step […] she found the solution”.*

(Nurse 8)

Respecting the patients’ timing is considered very important by nurses. Recognising that each patient reacts differently allows the nurse to engage the patient at the most appropriate time in order to make him/her feel like a person and not a number. Respecting the patient’s timing also means not forcing him to speak or to express his emotions until he decides to do so.

*“You don’t have to do everything at the same time, for example the information about the diagnosis, the information about treatment, all the arrangements, maybe all this at the same time is too much. So you also have to be able to that’s enough now and we’ll go back to that at a later time”.*

(Nurse 1)

### 3.5. Information Style

The communication process is considered very important to the nurses. The aspects that are taken into consideration are the information the patient has and his wish, or not, to receive further information on what is happening. It is crucial to know what the patient knows about his illness and what his sources of information are. People often try to fill in the information gap by consulting internet sites or learning from similar experiences in the family or among friends; this, at times, can cause fear and anxiety. It is important to discuss with people what they have read and to direct them to appropriate information sources. Alongside the desire to receive information, there can also be a desire not to be informed, and the nurse must take this into account. When conveying information, the nurses emphasised that the tone of voice is also important; maintaining a calm tone is crucial because it is precisely during the first moments of conveying information that the patient memorises, in the long term, what is being said.

*“Here is all that is information, so, making the patient understand what is going on […] so, here, informing with kindness, with calmness, with respect and with the means that he has at his disposal, that we have at our disposal […] the words that are said are the first photograph that the patient takes”.*

(Nurse 12)

Another aspect that nurses have emphasised concerns the content of the information. Explaining the benefits of treatment can promote the involvement of the person. Some nurses emphasise that besides treatment and the disease, it is also important to discuss with the person a very sensitive topic, which is pain management. In fact, patients often do not talk about this as they are culturally inclined to think that pain must be endured. In communicating with the person, giving practical examples is felt to be successful, allowing a greater understanding of what is being said and encouraging the person’s involvement.

*“I try to explain to them everything that surrounds it, that is, also what it means to manage pain, show him on a scale where pain may be unsupportable and what consequences it has, for example, the impact there could be on the treatment pathway, on the stay in the ward. Because maybe culturally we’re still saying ok, well if there’s a bit of pain, I can put up with it, so I won’t bother the nurses. I mean, instead what are the consequences of this and telling him about that”*

(Nurse 9)

### 3.6. Communication Strategies

Involving the patient also requires the nurse to get to know the person in front of him/her. Getting to know the other person means knowing his/her strengths, passions, and past experiences. Getting to know the person’s professional life also enables one to understand how he or she approaches problems and to give examples using the other person’s field of experience in order to make the information usable and comprehensible in the communication process.

*“The professional activity is very important because it allows me to understand if he has been a very practical person, to understand how he tries to reason […] hobbies are also very important because they give me a further clue and another thing […] if I have to explain something to him, I try to take him back to examples, maybe they’re a bit crazy because I don’t exactly know a certain type of work, but I try to get as close as possible to that reality he may have experienced”.*

(Nurse 3)

Another strategy used by nurses to increase the active involvement of patients is to motivate them. For instance, to find common ground that motivates them to commit to a certain aspect. Some also emphasise the importance of motivating the person even when faced with a worsening clinical condition and trying to create an alliance with him.

*“Motivating him, that is, finding a common ground that motivates one to take charge of an aspect or finding something in the patient that stimulates him”.*

(Nurse 14)

Nurses in the focus groups highlighted the importance of caregiver involvement, especially when patients need support. The family can also help to create a clearer clinical picture and help the nurse relate to the patient. The nurses emphasise that it is important to always consider the patient and his or her family as they form a single unit of care.

*“We see the patient and the family as a unit of care, so we take care of the patient and the people who are significant to the patient, that aren’t necessarily, maybe, close family but they can be other people. Because we realise that the disease impacts on the person and the people around him, and in this family circle we go to see where the resources are, or if there is a lot of suffering”.*

(Nurse 6)

Another strategy used by nurses is to resort to the testimony of experienced patients. The involvement of experienced patients is sometimes proposed by nurses to less engaged patients, while, at other times, it is the patients themselves who ask to speak to patients who have experienced a similar situation to their own.

*“She had specifically asked “but can’t I have the opportunity to talk to someone who has already gone through this experience, that’s had the same treatment that…” so we got together for a moment as a team and then we said “ok, let’s see what we can do about this request” and we thought of patients who had gone through the same process […] this young girl was contacted and we proposed […] that they meet, they talked then about a couple of weeks later this lady arrived for her treatment […] when they were saying about the side effects or “yes, yes she told me”, “yes, she told me that too, that it happens like that” and it was a kind of security for her”.*

(Nurse 7)

In order to foster patient involvement, nurses also spoke about “soft skills”, for example, helpfulness and perseverance, and also stressed the importance of active listening. These behaviours are even more useful when faced with uninvolved patients who, for fear or prior negative experiences, have put up barriers.

*“These barriers, from my experience, are due to fear. The patient doesn’t know, is afraid or has had a negative experience, and so doesn’t trust anyone in front of him. So first you have to, in my opinion, respect this barrier, be available, explain everything we are doing, make him part of it. In my experience, after some time in fact, the patient is the one who slowly opens up, I behave with him as I behave with everyone”.*

(Nurse 11)

### 3.7. The Care Team as a Patient Engagement Catalyser

In the focus groups, a theme emerged that encompasses the importance of working with colleagues and the care team to involve the patient. Communication within the team is crucial in order to share decisions and avoid discordant communication with the patient. The team is also important for discussing patient problems and finding solutions together. The experiences and skills of colleagues are valued. The team is also an important resource in terms of supporting its members when they are faced with difficult situations in relationships with patients, which can lead to emotional distress.

*“To always be able to rely on people who perhaps have more experience than you, but also perhaps not only clinical experience, maybe who have experienced other working realities, known other patients, worked, maybe, in other parts of the world with different cultures, and who can therefore always give you something more and can perhaps bring you solutions that you hadn’t thought of”.*

(Nurse 9)

## 4. Discussion

The aim of this study is to explore, from patients’ and nurses’ perspectives, what kinds of nurse-led, practice-based behaviour are more relevant to promoting patient engagement in cancer care.

For both patients and nurses, information is a key component in promoting patient engagement. Patients underlined that information is crucial to understand what is happening to them and, therefore, be capable of choosing what is perceived to be better for their clinical path. Partial (not exhaustive) or unclear information increases the worries of the patient. This is consistent with the literature [20,21] and underlines the strategic role of exhaustive and clear information and communication in the patient–nurse interaction to promote patient engagement in the care pathway.

Additionally, feeling free to ask questions is important for patients: in this regard, nurses play a key role because of their closeness with patients. In fact, where patients have sometimes not understood what physicians have told them, nurses become an important source of information, reiterating information in a simpler way. Tolotti et al. [22] found similar results in their study regarding oral chemotherapy drug education led by nurses. The use of medical jargon is a significant issue reported in the literature; this should be considered during the communication process [23]. Chegini et al. [24] highlighted the role of not-effective communication as a barrier to patient engagement.

Nurses underlined that the style with which information is delivered is also important. Firstly, it is important to actively listen to the patients and to understand what they know about their disease and their treatment pathway and any doubts they may have. Second, a calm, open and welcoming attitude is a key aspect that can increase patients’ trust in nurses and the clinical team. It is also important to ask patients for information about what they are sometimes not so comfortable sharing, such as pain, for example, as we found in our study. As reported in the literature, acute and chronic pain are important issues in cancer patients that can compromise their autonomy in daily life activities and worsen their quality of life [25,26]. Therefore, to make the patient involvement successful, this symptom must be recognised and treated by nurses.

In the study by Singh et al. [27], the authors emphasise that nurses should also adapt their communication styles to different patients. Furthermore, Chegini et al. [24] highlighted that bad communication can be a barrier to patient engagement. Strictly related to information, there is the patient–nurse relationship. Additionally, in findings within this study, patients and nurses have highlighted its importance. Patients consider nurses as an important reference point, a co-actor in their treatment process, and a person who can be trusted and relied upon. Similar results were found in the study by Tolotti et al. [22]. Additionally, for nurses, a relationship based on trust is essential to foster patient engagement, as reported in the literature [27,28,29,30].

Therefore, nurses should try to adopt certain strategies to create a relationship of trust with patients, who then tend to open up. The strategies implemented vary; there are those who are sincerely curious about what the patient says, those who do not stop at the door, and those who tend not to judge what is said by the patient but accept what is being said.

Patients also find the practice of active listening important in moments of despair when they need to vent to someone. The nurse knows that these moments are particularly valuable as they allow a trusting relationship to be established with the patient. The nurses try to make the patient understand that they are understood and that they should not feel inadequate. In the study by Padilla-Ruiz et al. [31], the use of active listening was shown to be effective in improving patient participation. Zucca et al. [32] highlighted the importance of asking the patient questions in order to establish an effective relationship.

To foster a good patient–nurse relationship, great attention must also be given to the setting in which the relationship takes place; it is vital to avoid interruptions in communication because it means taking care of the communicative process with the person. The language used can also prove useful in reducing possible communication distances between the nurse and the person.

For nurses, it is central to show availability and ensure the person feels free to express herself/himself, guaranteeing confidentiality. Maintaining continuity in the relationship with patients is also fundamental, as is leaving them free to choose the person with whom they wish to share their own or their family’s feelings and emotions.

For nurses, two further things that are important are to allow the patient to share their own emotions and to avoid blame.

Another essential finding for both stakeholders is the importance of person-centred care. For patients, it is important to be considered a person and not just a number or a disease. This result is consistent with the study by Mazzi et al. [33], where patients suggested that to reach such a goal, nurses must be interested not just in their disease but in all aspects of their life. This is crucial from a patient engagement perspective.

Nurses strive to adopt personalised care according to the patients they are dealing with. This can be achieved by using informal language that puts the patient and the nurse on equal footing, stepping outside the standards of care that nurses are familiar with, and tailoring care to the patient so that the patient feels understood and involved in his or her care. The significance of person-centred care in promoting patient engagement is underlined by several studies [4,21,32,33].

Nurses also emphasise the importance of teamwork and mutual support. For them, it is crucial that information given to patients is unambiguous and shared in order to avoid misunderstandings. This aspect is closely linked to the need for clear and truthful information, as required by patients. Teamwork is also essential to giving mutual support and dealing with those cases that are particularly stressful from an emotional point of view. Finally, the team is also useful because of the skill mix within it, as more experienced colleagues can show novice nurses what may be the best strategies to communicate with the patient, thus making them more involved. The importance of the team is also underlined by a recent review of the literature, which states that the prerequisites for active participation in shared decision-making are interdisciplinary teamwork, open communication, a good patient–healthcare professional relationship, a supportive environment and a mutual exchange of information [34].

Finally, the nurses suggested some strategies to enhance PE. A first strategy, which also relates to the nurse–patient relationship, is getting to know the patient in front of you in depth, identifying their level of psychological engagement with their own health in order to find the right personalised levers to favour his active involvement. The relevance of this strategy has been widely demonstrated in numerous psychology studies that have shown that the psychological and motivational profile of the patient is a parameter to be defined in order to personalise treatment initiatives and increase their effectiveness [2,35,36,37].

Among the strategies is the key involvement of the caregiver. In oncology and chronic diseases, the caregiver is an essential partner in the care pathway and can be a strategic ally for the healthcare team, as highlighted within the literature [38,39]. Caregiver engagement is seen as a crucial lever for the promotion of patient engagement, especially in situations where patient care is protracted over a long period of time; as a consequence, care in the person’s home environment becomes a value to be protected [40,41,42,43].

The nurses then suggest working on the patients’ motivation, considering the aspects that she/he considers most important, which can then encourage more active participation in the care pathway. This result is in line with two systematic reviews that have emphasised that interventions that act on motivation are the most effective in fostering patient engagement [12,13,44].

**Figure 1:**
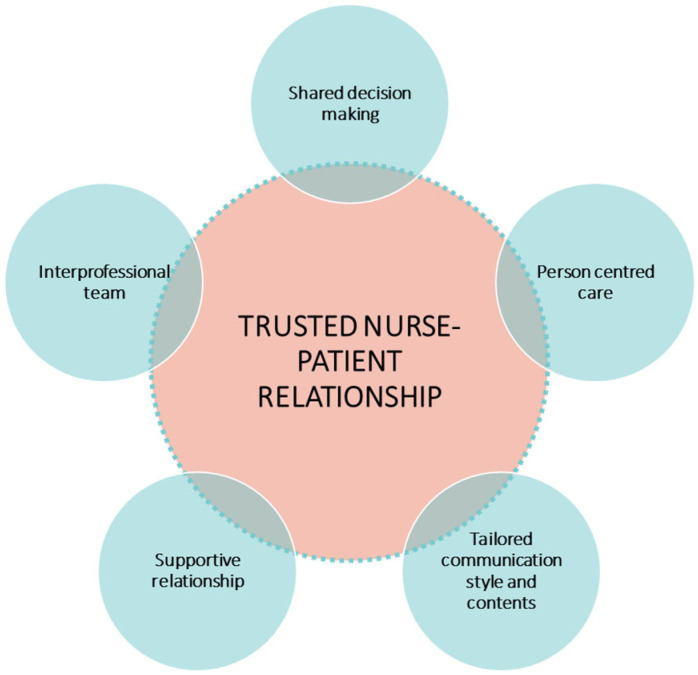
Patient engagement in oncology nursing practice: A conceptual diagram.

According to the study results, patient engagement in oncology nursing settings translates into some key elements that revolve around a central pivot (more specifically, both patients and nurses; see Figure 1). Patients and nurses underline the centrality of a therapeutic relationship based on trust as a necessary condition for the engagement of the person in his/her own care path. Around this pinnacle, five factors related to the nurses’ practice are pivotal to sustaining patient engagement in cancer care: (1) shared decision-making, which means eliciting patients’ values and goals, targeting the discussion of clinical options to those values and goals, and partnering with patients to make individualized decisions; (2) person-centred care, meaning an approach that fosters respectful, compassionate, culturally appropriate, and competent care that is responsive to the needs, values, beliefs and preferences of patients and their family members. Person-centred care shifts providers from doing something to or for the patient—where the health care provider’s perspective is dominant—to doing something “with them” in a true partnership; (3) tailored communication style and contents, meaning an information and educational process that takes into account the knowledge needs and the psychological adjustment of patients in every moment of his/her own illness journey; (4) supportive relationship, which is the emotional scaffolding function that patients expect from nurses besides the clinical actions; (5) interprofessional team, recognised as an organisational catalyser of the patient engagement process, where all disciplines work together and fully engage patients and those who support them; leadership on the team adapts based on patient needs.

### 4.1. Implications for Practice

Numerous studies have demonstrated the importance of the active involvement of cancer patients and those suffering from a chronic disease in the management of their disease as this improves quality of life and patient outcomes as well as reduces healthcare costs [8,45,46,47,48,49,50,51,52,53].

This study has allowed us to identify what nursing techniques and strategies are considered most important for patients and nurses in the oncology setting to promote patient engagement. To the best of our knowledge, this is one of the first studies that has investigated these aspects in the oncology setting. The suggestions that emerge from this study may be useful in developing effective interventions to foster patient engagement in this and other clinical settings with similar cultural and organisational characteristics.

From this study’s results, the authors are going to develop a tool capable of measuring these variables in terms of both their importance to the patient and the frequency with which these behaviours are put into practice by the nurse. The tool will be useful to understand what is important to the patients and how much this is realised by nurses, giving the possibility of implementing educational interventions, such as training courses, or modifying patient care, considering a more active involvement of the patient. This future line of research will engage patients/citizens, starting from the development of a patient-reported experience measure that is deeply rooted in the patient’s perspective, as suggested by recent reflection on the value of establishing collaboration with patients even in the clinical research process [54].

### 4.2. Implications for Research

Studies on patient engagement in oncology are few. This study can be a starting point to implement PE interventions by measuring them using the previously mentioned tool to see if they are effective in improving patient outcomes such as quality of life and active participation.

### 4.3. Limitations

This study has some limitations to consider. First, it was conducted within a single healthcare context. Local and cultural characteristics may have influenced the results, limiting their transferability to other contexts. However, many of the aspects that emerged from nurses and patients can also be found in the international literature, highlighting how strategies and techniques that promote PE are beginning to be transversal to different, even very different, contexts.

A second limitation is the fact that although efforts were made to maintain as much variability as possible, not all oncological diseases were investigated as saturation was quickly reached with the analysis of the interviews. It might be interesting in the future to conduct studies on specific groups of patients to assess whether the results identified with this study are confirmed or whether special needs related to the pathology emerge instead. Indeed, we know that oncological diseases have very heterogeneous courses and impacts on patients’ lives. Moreover, patients and nurses were selected according to their level of availability to openly discuss the topic of interest. It is possible that the ones more engaged also agreed to take part in the focus groups. Future studies should include a wider variability in terms of engagement levels in the study participants.

Finally, it was not possible to investigate the palliative care setting, although their involvement had initially been planned, due to the great difficulty of recruiting these patients in our context. It might be interesting to repeat the study in the future with this specific clinical population, for whom active involvement might be useful in terms of optimising quality of life.

## 5. Conclusions

The active involvement of patients in their disease is becoming increasingly important nowadays to promote better patient outcomes and reduce costs.

In this study, the importance of correct, sincere, and comprehensive information for patients to feel more involved emerges.

Great importance is given to the nurse–patient relationship, which must be based on trust and mutual respect. The nurse should have an active listening attitude to encourage the free expression of the patient of their concerns and doubts.

Both nurses and patients emphasised the importance of person-centred and personalised care, considering all aspects of the patient’s life and not considering the patient as just a number or a sick person.

The nurse is a key figure in active involvement due to his or her closeness to the patient and the therapeutic relationship that ensues. They give useful advice to patients, which helps them cope better with their condition.

Considering the above, a more systematic implementation of suggestions from the participants in this study is desirable for the future, avoiding that the strategies identified are only applied occasionally and on the basis of the goodwill of the individual practitioner; this is also emphasised by previous research [55,56,57].

## Figures and Tables

**Table 1:** Characteristics of the patients (n = 6).

Patients	Gender	Age	Oncological Disease
Patient 1	M	58	Leukaemia
Patient 2	F	65	Haematological cancer
Patient 3	M	75	Haematological cancer
Patient 4	F	43	Breast cancer
Patient 5	F	72	Multiple myeloma
Patient 6	M	84	Prostate cancer

**Table 2:** Characteristics of the nurses (n = 17).

Gender *	N (%)
M	4 (23.5)
F	13 (76.5)
**Age (Mean ± SD)**	42 (8.4)
**Clinical setting**	**N (%)**
Outpatient clinic	11 (64.7)
Radiotherapy (ambulatory)	2 (11.7)
Radiotherapy (ward)	1 (5.9)
Haematology (ward)	1 (5.9)
Clinical research	1 (5.9)
Palliative care	1 (5.9)
**Years of experience as a nurse (Mean ± SD)**	14.7 (7.4)
**Years of experience in cancer care (Mean ± SD)**	13 (6.3)

* M = male; F = female; SD = standard deviation.

## Data Availability

The data presented in this study are available on request from the corresponding author. The data are not publicly available due to privacy and institutional restrictions.

## Supplementary Materials

The following supporting information can be downloaded at: https://www.mdpi.com/article/10.3390/ijerph191811644/s1.
