# Supplementary material for: Patient Engagement in Oncology Practice: A Qualitative Study on Patients’ and Nurses’ Perspectives
Source: Int J Environ Res Public Health. 2022 Sep 15;19(18):11644. doi: 10.3390/ijerph191811644 (PMC9517681; doi:10.3390/ijerph191811644)
Supplement: Supplementary file 1 [file ijerph-19-11644-s001.zip › ijerph-1895628-supplementary.pdf]

International Journal of
Environmental Research and Public Health

MDPI

S1. Supplementary Materials: Patient's Interviews, and Nurse's Focus Groups Guides

Patients Interview Guide

Interviewer gives a brief description of the project aim and of the interview.

Opening Questions:

- Tell me about the stages of your care pathway (management in hospital and management of your care pathway at home).
- Which healthcare professionals motivated you in your care pathway? And which figures actively involved you in decision making during your care pathway?

Interviewer gives a brief description on the concepts of motivation and involvement

- Who provided you with information on the stages of the care pathway?
- Who gave you support and reassurance?
- Who provided you with clinical knowledge for symptom management at home?
- In your opinion, why is it important to involve (autonomy-decision making) a patient in the care pathway?
- To what benefit does involvement lead?
- Thinking back on your care pathway (in hospital, during therapy sessions, at check-ups), what role did the nurse play?
- In particular, what moments or situations with the nurse made you feel involved and active in your care?
- What episodes made you feel good?
- Thinking about the dialogues with the nurses, when did you feel most involved? And when or on what occasions did the nurse motivate you in the management of your illness?
- In addition to words, did the nurses' behaviour motivate or support you in the management of your illness?

Nurse's Focus Group Guide

- From your point of view, how do you define the active involvement of a patient in his or her care pathway?
- In your opinion, what benefits are derived from making patients an active part of the care pathway?
- From your point of view, what is the role and added value of nurses in promoting the active involvement of patients in the care pathway?
- Thinking about the patient's care pathway and its crucial moments (e.g. diagnosis, therapy, pain management...) what, from your point of view, particularly works to make patients actively involved? Can you give us an example of a moment/situation when you felt particularly effective in motivating him/her and tell us what you did/said?
- More specifically, what strategies or techniques do you consider particularly effective in informing patients and helping them to be active participants in decisions that affect them? Can you give us some practical examples? What do you say? What do you do?
- What strategies or techniques do you use to facilitate patients in learning effective behaviours in managing their disease? Can you give examples? What do you say? And what do you do?

• What strategies or techniques do you use to help him/her process the situation he/she is in? Can you give examples? What do you say? And what do you do?

- If you had to summarise the recipe for encouraging active patient involvement, what strategies would you suggest as being fundamental?

S2. Supplementary Materials

Table S1. Example excerpt from the thematic analysis—Main Theme: Effective information.

Meaning Units	Initial Codes	Sub Themes	Main Theme
"...in my opinion it's very important. In the sense that at least you make a patient aware of what's going to happen, of what I'm going to have to do, I'm going to have to endure or not … making me part of the situation, of the evolution of the situation, of what could be the problems …that is, of the whole treatment pathway" (Male aged 58)<br><br>"to Know what I have to do, what I have to endure or not. That's very important. Especially if it's good news (Female, aged 65)<br><br>"In my opinion it's really important, in the sense that at least a patient is made aware of what will or may happen" (Female, aged 72)	To be aware of the situation<br><br>To know what to have to do<br><br>To know actual and potential problems	To know is to understand	Effective information
"I expected that maybe there would be more involvement, saying like today we're going to irradiate this, today we are going to irradiate this other, we've finished with this part and we're going to start with the other. The only information I was given by the Doctor was that the first part of the treatment lasted 20 days or 25, was about the lymph nodes that had already	Expecting more involvement in the pathway	To be engaged through information	

formed, bombard the different lymph nodes in the surrounding areas. And the last 15 sessions I think, 15 or 14, for the prostate directly […] if I'd have had some more information, it would increase my involvement. We need information!" (Male aged 84)<br><br>"If I have any questions, they know how to respond or they go and ask the doctor [...] […] (Male, aged 85)<br><br>"... if I'm at home I need ask something, I just call here and they also give me the answer" (Female, aged 72).	Getting answers<br><br>To be able to ask even from home		
"They always treated me very well, motivated, said the right things at the right time eh…very positive experience …" (Female, aged 43)<br><br>"I was afraid, of something new, so he slowly explained to me and said "do it like this, it becomes easier this way" (Male, 72 aged)<br><br>"At home you know I was afraid to touch it (wound), because it hurt, so he said "start moving ", otherwise I was always sitting on the chair at home and I was there, he said to me" take the stairs go for a walk ", because I wasn't going out anymore" (Male, 75 aged).	To choose the right information and time to inform the patient<br><br>To give appropriate information<br><br>Choose the information that you need	The right time and the right content	

Table S2. Conceptual framework outlining the main themes and subthemes from the interviews with patients.

Main Theme	Sub-Themes
Effective information	To know means to understand something<br>To be engaged through information<br>The right time and the right content
Having the opportunity to choose	The role in decision making<br>Lack of information<br>To revisit what has been said<br>Trust in nurses
Being considered as a person	Not feeling like a number<br>Being seen as a person
Trusted relationship with nurses	Nurse is a co-actor in the patient care process<br>To feel the nurse is interested
Receiving support and advice".	Helping people to cope with their difficulties<br>To spur the person on
